# Unique Names in China: Insights From Research in Japan—Commentary: Increasing Need for Uniqueness in Contemporary China: Empirical Evidence

**DOI:** 10.3389/fpsyg.2020.02136

**Published:** 2020-09-29

**Authors:** Yuji Ogihara

**Affiliations:** Faculty of Science Division II, Tokyo University of Science, Tokyo, Japan

**Keywords:** name, uniqueness, cultural change, individualism, China, Japan, need for uniqueness, culture

## Abstract

By comparing naming practices between China and Japan, I propose three suggestions on Cai et al.'s (2018) Study 2, which examined historical changes in baby names in China. Their study found that the average daily frequencies of Chinese characters used in baby names decreased between 1950 and 2009. The authors concluded that unique names increased for this period and suggested a rise in the need for uniqueness and individualism in China. However, there are three questions that have remained unanswered. First, did the Chinese characters that were used in names indeed become more unique over time? Second, did the number of Chinese characters in names increase over time? Third, did the reading (pronunciation) of names become more unique over time? Answering these three questions would further increase the validity and impacts of the article and contribute to a better understanding of cultural changes in China.

Cai et al. (2018) examined temporal changes in need for uniqueness (NFU) in China. Specifically, in Study 1, they measured NFU among participants aged between 13 and 62 and found that age was negatively correlated with NFU. In Study 2, they collected 10 first (given) names of babies per year for the 60 years between 1950 and 2009 (i.e., 600 names in total) and found that recent baby names tend to have infrequent Chinese characters. They concluded that both studies suggested a rise in NFU and individualism in China, which is consistent with findings in other research (e.g., Hamamura and Xu, 2015; Zeng and Greenfield, 2015; Ogihara, under review).

In this article, due to space limitations, I focus on Cai et al.'s (2018) Study 2, which investigated temporal changes in baby names in China, and propose three suggestions. I have conducted research on unique names (e.g., Ogihara, 2015; Ogihara et al., 2015; Ogihara and Ito, 2020) and cultural changes in Japan (e.g., Ogihara et al., 2016; Ogihara, 2018b; for reviews see, Ogihara, 2017, 2018a). China and Japan, both of which are located in East Asia, share many aspects, such as language (e.g., the use of Chinese characters) and value (e.g., yin-yang thinking style). Thus, by comparing characteristics and trends in unique names between China and Japan, I propose three suggestions.

## 1. Did the Chinese Characters that Were Used in Names Indeed Become More Unique Over Time?

Cai et al. (2018) investigated historical changes in the average frequency of Chinese characters and suggested “the increasing prevalence of unique names” (p. 4). They also stated that “Study 2 found that Chinese people have used increasingly unique names for their children over past 50 years” (p. 4)^1^.

However, does the result truly mean that the rate of unique names increased? Whether a name is unique or not is usually determined by how similar/different it is from other names in the same birth cohort (e.g., Twenge et al., 2010; Ogihara et al., 2015). If there are few or no other babies with the same name, it is (regarded as) unique. In contrast, if there are many babies with the same name, it is not a unique name. To identify the uniqueness of each character, the authors used a general dictionary to assess its frequency of use (the Modern Chinese Character Frequency of Use Dictionary), but this dictionary was not specialized for baby names. A character that has a low frequency in daily use does not necessarily have a low frequency in baby names. The letters may be infrequent in daily life in general, but they may be frequently used for baby names. Thus, it is possible that parents increasingly used Chinese characters that were infrequent in daily life but were common for names.

To answer this question, it would be better to conduct further analyses. Making a new dictionary of Chinese character frequency for baby names would be desirable, but 600 names are insufficient to make a valid dictionary. Further, it seems difficult for the authors to add a sufficient number of baby names. However, it would be possible to conduct some analyses using data the authors already collected. For example, the authors have 100 names for each of the six cohort groups. Thus, it is possible to count how many Chinese characters were duplicated in each group and analyze their temporal changes. If Chinese characters indeed became unique, the duplication rates would decrease over the period. It would also be possible to focus on variations of Chinese characters in each of the cohort groups and analyze their historical changes. Because the sample sizes are not sufficiently large, these analyses may not yield a clear trend. However, it would be valuable to add these analyses.

## 2. Did the Number of Chinese Characters in Names Increase Over Time?

There is no explicit regulation about the usage of Chinese characters in China, unlike in Japan (Table 1). Therefore, it makes sense that using unique Chinese characters would be an efficient strategy for giving a unique name to a baby in China.

**Table 1:** Summary of restrictions in giving names to babies in China and Japan.

**Domain**	**China**	**Japan**
Variation of Chinese characters	–	○
	• The Chinese government has no formal rules to restrict the use of Chinese characters for baby names. • It is recommended that very rare Chinese characters should not be given to babies, but it is not an official regulation.	• The Japanese government restricts the number of Chinese characters that can be used for baby names. • The number of Chinese characters is approximately 3,000.
Pronunciation of Chinese characters (paring between writing and reading)	○	–
	• It depends on what the character is, but most Chinese characters have only one pronunciation.	• Most Chinese characters have multiple pronunciations. • Moreover, any pronunciation can be given to a Chinese character for baby names even if that pronunciation is not used in daily life.
Number of letters	–	–
	• There is no explicit restriction regarding the number of letters for baby names.	• There is no explicit restriction regarding the number of letters for baby names.

*In the domain where there is a restriction, variations of names are inhibited, making it difficult to express uniqueness. Without restrictions, it is relatively easy to give rare, uncommon, and novel names. It seems that people tend to express uniqueness in the domains where there are no restrictions*.

However, is using uncommon Chinese characters the only way to give unique names? Another possible way is to give longer names. The authors stated that “[t]ypically, a Chinese given name consists of either one or two characters based on the preference of the child's parents or elderly family members” (p. 3–4). This “typical” practice of giving one or two characters may have gradually changed over time. Deviating from typical practice is one way to express uniqueness. If names have more than two characters, they are more likely to be unique because they are different from typical names. Moreover, as the number of characters in a name increases, the name has a lower probability of being duplicated with other names, thus increasing the probability of being unique. Therefore, the number of letters in names may have increased^2^.

Furthermore, within the typical practice of giving names with one or two characters, people may have become more likely to choose names with two characters than to choose names with one character because it decreases the probability of duplicated names and increases the probability of unique names. This change may also have led to the increase in the characters used for baby names.

The authors already computed and used the corresponding variable in the multiple regression analysis [“name length (i.e., the total number of characters in the given name)”; p. 4]^3^. Thus, it should be easy to examine these two hypotheses.

## 3. Did the Reading (Pronunciation) of Names Become More Unique Over Time?

The authors focused on the writing of names (Chinese characters) and found that the average frequency of Chinese characters in daily life in general decreased over time.

However, what about the reading (pronunciation) of names? To fully understand temporal changes in baby names in China, it would be desirable to reveal the other aspect of names. It is possible that Chinese characters became more unique but that pronunciations became less unique. In fact, in Japan, the pronunciations of names became more unique, while the Chinese characters used in names became less unique (Ogihara et al., 2015).

If the authors have data for writing of names but do not have data for reading of the names, it would be possible to code the names. It depends on what the character is, but most Chinese characters have only one pronunciation in China (Table 1). For Chinese characters with multiple pronunciations, coders who are blind to the hypothesis can estimate the most probable pronunciations, and the estimations can be confirmed among the coders. Analyses can be conducted for both the case when the names with multiple pronunciations are included and the case when they are excluded. Then, the authors can confirm whether the results obtained from these two analyses are consistent.

## Conclusion

I propose three suggestions that would further increase the validity and impacts of the article (Cai et al., 2018). I hope these comments will contribute to a better understanding of the historical changes in baby names and their underlying psychological/cultural trends in China.

## Author Contributions

The author confirms being the sole contributor of this work and has approved it for publication.

## Conflict of Interest

The author declares that the research was conducted in the absence of any commercial or financial relationships that could be construed as a potential conflict of interest.

## Footnotes

^1^In Figure 2, the authors showed character frequencies averaged for each of the six birth cohorts. Although it is unclear what the error bars indicated (e.g., standard deviation, standard error, confidence interval), the variances of the frequencies decreased over time. This may indicate that the way of giving unique names by using uncommon Chinese characters became more shared.

^2^Even if this hypothesis was already examined by other researchers, it is still important to replicate the finding by analyzing an independent dataset. That would further increase the validity of the finding.

^3^Because naming practices differ across ethnic groups (e.g., Stojcic et al., 2020), ethnicity should also be controlled for with the other confounding variables (gender, name length).
